# Supplementary material for: Keeping in step with the young: Chronometric and kinematic data show intact procedural locomotor sequence learning in older adults
Source: PLoS One. 2022 May 3;17(5):e0266733. doi: 10.1371/journal.pone.0266733 (PMC9064075; doi:10.1371/journal.pone.0266733)
Supplement: Attachment — Submitted filename: Johannsen_Bollheimer_responses_to_comments_b.docx [file pone.0266733.s001.docx]

Dear Dr. King and Reviewers,

we kindly appreciate your time for editing and reviewing our submitted manuscript. We have considered your feedback and suggestions and have addressed them as best as possible. In the following overview, we have listed your single recommendations and our responses and corresponding changes made to the manuscript.

Editor comments

1. “While I agree with Reviewer 2 that the intro is well-written, it is my opinion that it would be beneficial to considerably reduce the introduction in length. For example, the SRTT paradigm is a common protocol and can be succinctly and clearly introduced in a sentence or two. Similarly, it would be advantageous to only introduce those concepts that are directly relevant to the current paper (e.g., different sequence representations, chunking, etc. were not assessed here and thus do no warrant such an elaborate introduction).”

*Response: the introduction was reduced in length by removal of those paragraphs relating to chunking and sequence representations including a short section referring to research conducted in monkeys.*

2. “A similar comment can be applied to the abstract. Not necessary here to mention the number of blocks or the specific block numbers used in the computation of learning.”

*Response: the abstract was slightly shortened by the removal of methodological information but also received an edited conclusion.*

3. “It is unclear as to how results from Experiment 1 were used for the power computation for Experiment 2. Experiment 1 did not assess age differences; and, ultimately, data from the available literature was used for the power comp (Howard & Howard). Regardless, the two groups then exceeded this desired sample size (27 and 22 in the two groups as opposed to 16 each). So there appears to be no data-driven justification for the precise sample in Experiment 2.”

*Response: the comment in the methods section of experiment 2 regarding power calculations for sample size estimation based on aging effects reported in the literature has be revised and reformulated. We still have to refer to the available literature but restrict our expectations to large effects only. This has also been considered in the general discussion section.*

4. “The following information (applicable to both experiments) is better fit for the data processing / reduction section: “.. the first trial of each block was excluded from analysis. In addition, for all outcome parameters except error proportions, error trials and the trials following an error trial were excluded to avoid the influence of post-error slowing on latencies”

*Response: these lines have been moved into methods sections of both experiments respectively.*

5. “If I understood correctly, the measure T-PWS was not valid for mats 3 and 4. Is this correct? If yes, this no longer seems like a useful dependent measure given that the large number of trials that were discarded.”

*Response: all mentioning of force plate recordings and derived preparatory weight shift outcome parameters has been removed from the manuscript.*

6. “For experiment 2, it would be advantageous to indicate whether the sequence specific learning effects (middle columns; Table 3) are significant WITHIN each age group. The last column collapses across the two groups and shows significant learning effects, but this appears to benefit from the large n (collapsed across young and old) and effects that are disproportionately larger in young (although not sig. different from older). In brief, it does not appear that the older adults exhibit significant learning per se on many metrics. This information would be of interest. Note that this does not take away from the main between-group comparisons, but would be worth specifying nonetheless.”

*Response: separate t-test for within-group significance of learning effects have been calculated and added to Table 3.*

7. “I leave this up to the authors but I am not convinced the methods of the two experiments cannot be presented within a single methods section followed by results of the two experiments.”

*Response: in an earlier draft of the manuscript before submission the methods sections in Experiments 1 and 2 were unified. However, the authors decided to split up the methods sections and prefer to keep this separation.*

Additional requirements

8. “We note that the grant information you provided in the ‘Funding Information’ and ‘Financial Disclosure’ sections do not match. When you resubmit, please ensure that you provide the correct grant numbers for the awards you received for your study in the ‘Funding Information’ section.”

*Response: the grant information in funding information and financial disclosure statements have been matched.*

9. “We note that you have provided funding information that is not currently declared in your Funding Statement. However, funding information should not appear in the Acknowledgments section or other areas of your manuscript. We will only publish funding information present in the Funding Statement section of the online submission form.”

*Response: the funding information has been removed from the acknowledgements section.*

Reviewer 1 comments

10. “The locomotor SRTT has three different step types (forward step, backward step, foot on same spot), two of which (forward, backward) could have followed a step by the same foot, or by the opposite foot. It seems that these five conditions (forward/same foot, forward/different foot, backward/same foot, backward/different foot, same spot) present different postural and balance concerns and difficulty, which could result in different challenges to older adults. The finger movement SRTT literature demonstrates aging effects increase for higher-order sequence information and other forms of increased complexity (e.g., Howard et al., 2004, Psychology and Aging; Bennett et al., 2007, Journals of Gerontology - Series B); it may be then that in a locomotor SRTT, aging effects become apparent for the more difficult/complex movements. Did step type have any impact on sequence learning, or interact with age effects on sequence learning?”

*Response: we preformed this suggested analysis and included it in the methods, results and discussion sections of Experiment 2 and the general discussion. Although, stepping complexity effects were found, we observed no interactions with age group.*

11. “Line 105: The quick mention of generalization feels out of place in the paragraph on chunking; I would recommend separating these concepts, and perhaps expanding on the generalization point.”

*Response: the paragraphs referring to chunking, generalization and representations have been removed from the introduction.*

12. “Line 257: What is meant by “Each participant received an individualized set of three randomized sequences”? I presume that this means the sequences used had the same archetypal format across participants (i.e., A-C-D-B-A-C), but that the mapping of [1,2,3,4] to [A,B,C,D] is what was randomized three times for each participant. If this is instead referring to a loop within the random condition, that should be clarified, and potential predictability driven by that loop would need to be addressed in all analyses. Could you please clarify this point?”

*Response: this statement has been rephrased to express that each participant received completely randomized blocks 1, 2, and 9. At the start of the experiment the random sequence of elements were generated anew for each participant.*

13. “Line 309-310: This sentence is unclear; I believe it is missing a phrase (“In addition, for response latencies but not ???, error trials and…”)”

*Response: this sentence has been rephrased to clarify its meaning.*

14. “Table 1: It would be good to include the random condition means (Block 9) in this Table for direct comparison with the sequence condition means. Also, assumedly EP means error proportion, but that is not specified- and if the units are % it cannot be a proportion.”

*Response: the random condition means move been included in the Tables 1 and 3. Also, “error proportions” have been rephrased as “percent error” in the entire manuscript.*

15. “Line 381: It is good that participants with severe hearing impairments were excluded, but what about non-severe aging-related hearing loss? Given that the cues were auditory, even moderate presbycusis could cause additional cue processing time for older adults that would affect responses in a manner unrelated to movement planning. Were audiometric thresholds obtained for older adults, or was hearing assessed in any other objective manner?”

*Response: the description of the recruitment procedure for the older adults in Experiment 2*

*has been reformulated. Although participants were contacted via a hospital database, any audiometric thresholds were not assessed. Nevertheless, all participants were able to understand the auditory target cues acoustically.*

16. “Figure 3: What do the red and blue traces in the CoP position time series plots differentiate? I am assuming ML and AP, but it should be labelled. Also, perhaps this is just an aspect of the coordinate system used for measurements, but if this Figure illustrates a forward step by the left foot, why are left foot displacement and velocity negative?”

*Response: the figure has been edited to that a forward foot displacement results in a flipped, positively increasing trajectory. The original figure showed the raw data in the kinematic coordinate frame, which had negative in the forward stepping direction.*

17. “Line 471: Though the Chi-squared test did not reach significance, the descriptive statistics suggest an aging effect here. Moreover, for older adults the full range of number-of-elements-reported is given, whereas only the lower bound is mentioned for young adults. Did older and young adults differ on number of sequence elements correctly reported?”

*Response: the average number of elements in a correct sequence was calculated and tested between both age groups. No differences were found. This was included in the results section.*

18. “Line 496: After stating the lack of age x sequence condition interaction, it would be helpful to report the lower bound of the observed p-value (e.g., p > 0.2). This comment applies to all such statements of null effects intended to be meaningful in the Results sections.”

*Response: for all observed non-significant p-values, the F or t statistics, p values and partial eta-squared have been included in the manuscript.*

19. “Line 533: What was the reasoning for considering the T-PWS, T-STEP, and T-TARGET measures to be the main movement parameters? If these are the parameters of primary interest, why were multiple other measures included in analyses too?”

*Response: an explanation was added to better explain the reasoning for the inclusion of secondary movement parameters.*

20. “Line 550: The comma between “participants” and “who” disrupts the meaning of the sentence. There are multiple odd commas like this throughout the manuscript- these should be identified and removed.”

*Response: we have probably overgeneralized german punctuation rules concerning relative clauses and inserted superfluous commas. We tried to identify and remove those.*

21. “Line 553: Would be good to remind here that the reason T-STEP and T-PWS are not mentioned is that those measures were not possible in Experiment 1. You mention this later (line 580), but it would be useful here.”

*Response: a reminder has been added that the T-STEP parameter etc. were not available in Experiment 1.*

22. “Line 562: I don’t recall seeing the T-PWS sequence effect being reported as marginal in the Results. I see in Table 3 the p-value for that comparison was 0.17, which is a stretch for including as marginal- the authors do hint here though that the effect was more reliable in young adults alone, which is not reported. Given the lack of age x sequence interaction though, such a breakdown would not be a licensed comparison. This claim should either be better defended, or removed.”

*Response: this statement has been removed.*

23. “Line 601: The given interpretation that locomotor sequence learning may be less susceptible to aging decline than finger movement sequence learning is certainly reasonable. An alternate interpretation the authors may want to consider is that the aging effects reported in the finger movement literature may instead reflect a relative expertise of young adults for finger sequence movements, rather than an aging-related decline, and that this aging-related difference in expertise is not observed for stepping movements.”

*Response: a statement has been added to the discussion which considers a possible specialization of younger adults in finger activities with respect to the interpretation of aging effects in the literature.*

Reviewer 2 comments

24. “It is unclear whether accuracy was emphasized to participants. What instructions were participants provided about the speed and accuracy of their movements? This may affect how quickly and accurately they respond to the auditory stimuli.”

*Response: in order to clarify the instructions regarding emphasis on speed or accuracy we moved this information to a more prominent position.*

25. “Was a recall or recognition test used to assess whether participants were able to determine that there was a sequence in the stimuli? It seems so, as some results are described, but the procedure and questions asked should be described in the methods.”

*Response: the description of the procedure for the recall and recognition has been rephrased with additional detail.*

26. “What were the results of the paired t-tests in Experiment 1 and the ANOVA in Experiment 2? The methods describe these analyses, but they are not reported in the results.”

*Response: we reformulated sentences in the methods and results sections to signpost the results of paired t-tests and ANOVA better in both experiments.*

27. “What is the purpose of the Pearson correlations? What part of the research question were they trying to answer?”

*Response: we added further explanations of the purpose of the Pearson correlations.*

28. “As older adults may make more anticipatory postural adjustments, how were they accounted for with respect to the postural adjustments all participants made to get ready to take a step? Was there any difference in these augments between the older and young adults?”

*Response: as all force plate measures have been removed from the manuscript a difference between old adults and young regarding APAs is no longer reasonable in the context of the experiments. It is an interesting question indeed but would need a follow-up study (currently in progress).*

29. “The authors statement that, “Surprisingly, despite decreased functional reserves and reduced locomotor stability that occurs with aging (i.e. chronological age), it had no obvious influence on the acquisition of the stepping sequence in terms of any latencies, movement parameters or error rates” (lines 594-596) is a bit confusion. It seems from Figure 4 that older adults were overall slower across all blocks and made more errors. Perhaps the ANOVA results would clarify this.”

*Response: in order to improve the clarity of this statement this section has been edited slightly.*

30. “The conclusion seems a bit overstated and broad. For example, how can this task be used as a clinical tool? Without assessing the task on a clinical population, it is unclear which parameters would be valuable for assessing a clinical population, especially since the authors did not find any significant differences between the older and young adults.”

*Response: a paragraph has been added to the general discussion section to suggest possible clinical applications of the paradigm.*

31. “This is a minor stylistic suggestion. There are many abbreviations used in the manuscript that disrupt the flow of the paper. Some abbreviations are defined, but never referred to again (e.g., AR in line 157) that can be taken out.”

*Response: we reduced the number of abbreviations.*
