# Supplementary material for: Keeping in step with the young: Chronometric and kinematic data show intact procedural locomotor sequence learning in older adults
Source: PLoS One. 2022 May 3;17(5):e0266733. doi: 10.1371/journal.pone.0266733 (PMC9064075; doi:10.1371/journal.pone.0266733)
Supplement: Attachment — Submitted filename: Johannsen_Bollheimer_responses_to_comments_rev2.docx [file pone.0266733.s002.docx]

Dear Dr. King and Reviewers,

once again, we like to express our gratitude for editing and reviewing our submitted manuscript. We have taken the additional feedback and suggestions seriously and addressed them in the manuscript accordingly. In the following overview, we have listed each recommendation and our response and the changes made to the manuscript.

Reviewer 1 comments

1) The analysis breakdown by step complexity for Experiment 2 is welcome, and fully addresses my concern that more complex movements might reveal aging-related impacts on sequence learning. The non-age-dependent effects of step complexity on kinematic measures reported for Experiment 2 are also informative for future work that may use similar designs. However, it is unclear why the complexity analyses were only performed for Experiment 2; there is no obvious reason why similar analyses could not have also been performed on Experiment 1, which would give a sense of reliability to the complexity effects. This would also make sense conceptually, as Experiment 1 is introduced as a way to validate the approach used in Experiment 2, so it is unclear why an unvalidated additional analysis is introduced for Experiment 2.

*Response: we have now performed and included the analysis of the effect of step complexity for Experiment 1. This change required alterations to the methods sections of both Experiments for the sake of consistency. Similarities between both experiments are discussed in the summary section of Experiment 2.*

2) A power analysis based on data from Experiment 1 indicated that n = 9 was sufficient for detecting large within-group response latency effects, and previous work used ns between 15–40 to demonstrate age effects. However, these points are then used to justify a sample size of 22 as an a priori target for detecting large age effects across a number of dependent variables; it is not clear how those two data points support that conclusion. It seems that 22 was the minimum group n, and the power justification is more post-hoc than a priori. Wouldn’t it make more sense then to do a post-hoc power calculation instead and state what effect sizes you had power to detect?

*Response: we have followed the reviewer’s suggestion and modified the paragraphs in the methods section of Experiment 2 in terms of a discussion of a post-hoc power analysis. In the General discussion section we are also now referring to the power issue.*

3) For Experiment 2, why were ANOVAs used only for T-STEP and T-TARGET? Mixed ANOVA seems an appropriate statistical choice for all dependent variables analyzed here.

*Response: thank you for pointing this out. This statement is clearly mistaken, it represented an earlier state of the draft, and has been corrected. Of course, mixed ANOVAs were also applied for the remaining dependent variables.*

4) Figure 1: The caption here suggests that the reflective markers for the optoelectronic system were worn for both Experiment 1 and Experiment 2, but if I understand correctly the optoelectronic system was only employed for Experiment 2. If that is correct this caption needs to reflect that.

*Response: we have extended the caption of this figure to state that the markers were only used for Experiment 2.*

5) Line 352, what is meant by “I presentation of the auditory stimuli”?

*Response: this was a typo and has been corrected.*

6) Line 371, “was defined” seems extraneous.

*Response: has been removed.*

7) Line 382, “mediolateral” is not in Figure 3 and doesn’t need an acronym defined here.

*Response: has been removed.*

8) Lines 384 – 408, this first paragraph in the Experiment 2 Statistical Analysis section would benefit from being broken into 2-3 smaller paragraphs.

*Response: this section has been broken down into smaller paragraphs.*

9) Lines 476 – 477, it is claimed that Table 3 contains the t-test and ANOVA results for all DVs, but Table 3 only contains t-test results.

*Response: has been corrected by removing the reference to any ANOVA results*

10) Table 3: STEP-DUR t-test results for each group are not significant, but bolded.

*Response: has been unbolded.*

11) Table 4: Bolding is inconsistent in Table 4 too. Unless alpha in Table 4 was 0.001? If so, that should be specified somewhere.

*Response: we have stated in the statistics section now, that a more conservative significance criterion was chosen.*

12) Table 5: What do the italics indicate? Also, please define acronyms in Table caption.

*Response: the italics only served a purpose during preparation of the manuscript and have been removed. Also, we explained the acronyms in the table.*

13) Lines 600 – 606, Although the group differences in explicit knowledge did not reach

significance, the group means were consistent with the extant literature. So, I would not lean too hard on the current null finding as counter-evidence to prior demonstrations of age differences in explicit sequence knowledge during the SRTT.

*Response: we have softened our conclusion accordingly and addressed the issue of statistical power here.*
